# Deep-Time Phylogenetic Clustering of Extinctions in an Evolutionarily Dynamic Clade (Early Jurassic Ammonites)

**DOI:** 10.1371/journal.pone.0037977

**Published:** 2012-05-25

**Authors:** Clotilde Hardy, Emmanuel Fara, Rémi Laffont, Jean-Louis Dommergues, Christian Meister, Pascal Neige

**Affiliations:** 1 Laboratoire Biogéosciences, UMR CNRS 6282, Université de Bourgogne, Dijon, France; 2 Département de Géologie et de Paléontologie, Muséum d'Histoire Naturelle de Genève, Geneva, Switzerland; Ecole Normale Supérieure de Lyon, France

## Abstract

Conservation biologists and palaeontologists are increasingly investigating the phylogenetic distribution of extinctions and its evolutionary consequences. However, the dearth of palaeontological studies on that subject and the lack of methodological consensus hamper our understanding of that major evolutionary phenomenon. Here we address this issue by (i) reviewing the approaches used to quantify the phylogenetic selectivity of extinctions and extinction risks; (ii) investigating with a high-resolution dataset whether extinctions and survivals were phylogenetically clustered among early Pliensbachian (Early Jurassic) ammonites; (iii) exploring the phylogenetic and temporal maintenance of this signal. We found that ammonite extinctions were significantly clumped phylogenetically, a pattern that prevailed throughout the 6.6 Myr-long early Pliensbachian interval. Such a phylogenetic conservatism did not alter – or may even have promoted – the evolutionary success of this major cephalopod clade. However, the comparison of phylogenetic autocorrelation among studies remains problematic because the notion of phylogenetic conservatism is scale-dependent and the intensity of the signal is sensitive to temporal resolution. We recommend a combined use of Moran's *I*, Pearson's *ϕ* and Fritz and Purvis' *D* statistics because they highlight different facets of the phylogenetic pattern of extinctions and/or survivals.

## Introduction

The disappearance of species has become a major scientific and societal concern over the last decades. On the one hand, conservation biologists are devoting much effort to understand current extinctions and their potential consequences. On the other hand, palaeontologists contribute to the debate by putting the current erosion of biodiversity into a deep-time perspective [1]. These two approaches to the study of extinction, despite using different data and scales, are currently getting closer as they both increasingly incorporate the same factor: phylogeny (*e.g*. [2], [3]). In fact, this conceptual and methodological convergence has already revealed that most current extinction risks and past extinctions are phylogenetically non-random: taxa in some lineages are consistently more extinction-prone than others [2]–[9]. This phylogenetic clustering is frequent at several spatial and temporal scales, suggesting that extinction-related key traits (or combination of traits) are themselves phylogenetically conserved [2], [3], [6], [10], [11]. The most frequently identified factors contributing to extinction are large body size, narrow ecological tolerance, limited dispersal ability, or high trophic level. Not only do these life-history traits often covary, but they are also linked to other macroecological features such as small geographic range size and low abundance (e.g. [5], [6], [10], [12]–[14]). Although all these factors do not directly evolve along the branches of the phylogeny, they often tend to be phylogenetically non-random. Such a pattern is interesting because it may help to identify the ultimate causes of extinction and to evaluate the complex impact of extinction on the loss of evolutionary history [2], [10]–[18].

From this recent endeavour, two observations can be made. The first one is that there is no consensus on how to quantify the phylogenetic signal of extinction (which is treated as a binary variable). Proposed techniques involve the Moran's *I* autocorrelation coefficient [19]–[21], the Pearson's correlation coefficient *r* [3], or indices using the sum of sister-clade differences [10], [22], [23]. These approaches differ in their exploitation of taxonomic/phylogenetic information and in their ability to yield comparable measures of phylogenetic signal strength.

The second observation is the dearth of quantitative studies dealing with the phylogenetic distribution of extinctions in the geological past (but see [2]). To our knowledge there is currently no high-resolution study on that subject, and evolutionarily dynamic clades (*i.e*., clades with high rates of taxonomic turnover) have not been investigated in that perspective. The fossil record offers a unique opportunity to assess the variation of the signal through time based on actually observed extinctions and survivals rather than on extinction risks [2], [3], [11], [17].

Here, we introduce a species-level dataset on ammonites (extinct cephalopods) for the early Pliensbachian stage (Early Jurassic, 189.6Ma–183Ma, [24], [25]). During that deep-time interval characterised by an important warming of seawaters [26], [27], marine organisms showed background rates of extinction, a marked provincialism, and significant variations in both diversity and morphological disparity, especially in the western part of the Tethys Ocean [28]–[33]. Our dataset has one of the best temporal resolutions available for such a remote geological interval, and it concerns a diverse clade with an excellent fossil record. Indeed, early Pliensbachian marine deposits are widely exposed in Europe and North Africa, and they yield abundant ammonite assemblages that have been extensively studied since the nineteenth century, chiefly for dating stratigraphic successions [32].

We then briefly review the approaches used to quantify the phylogenetic selectivity of extinctions and extinction risks, and we apply them to address the following questions: are early Pliensbachian ammonite extinctions phylogenetically clustered? If so, what are the phylogenetic levels concerned? How does that pattern vary over geological time and when the temporal resolution changes? To what extent does the survival of species correlate with the clustering of extinctions?

## Materials and Methods

### Datasets

**Figure 1:**
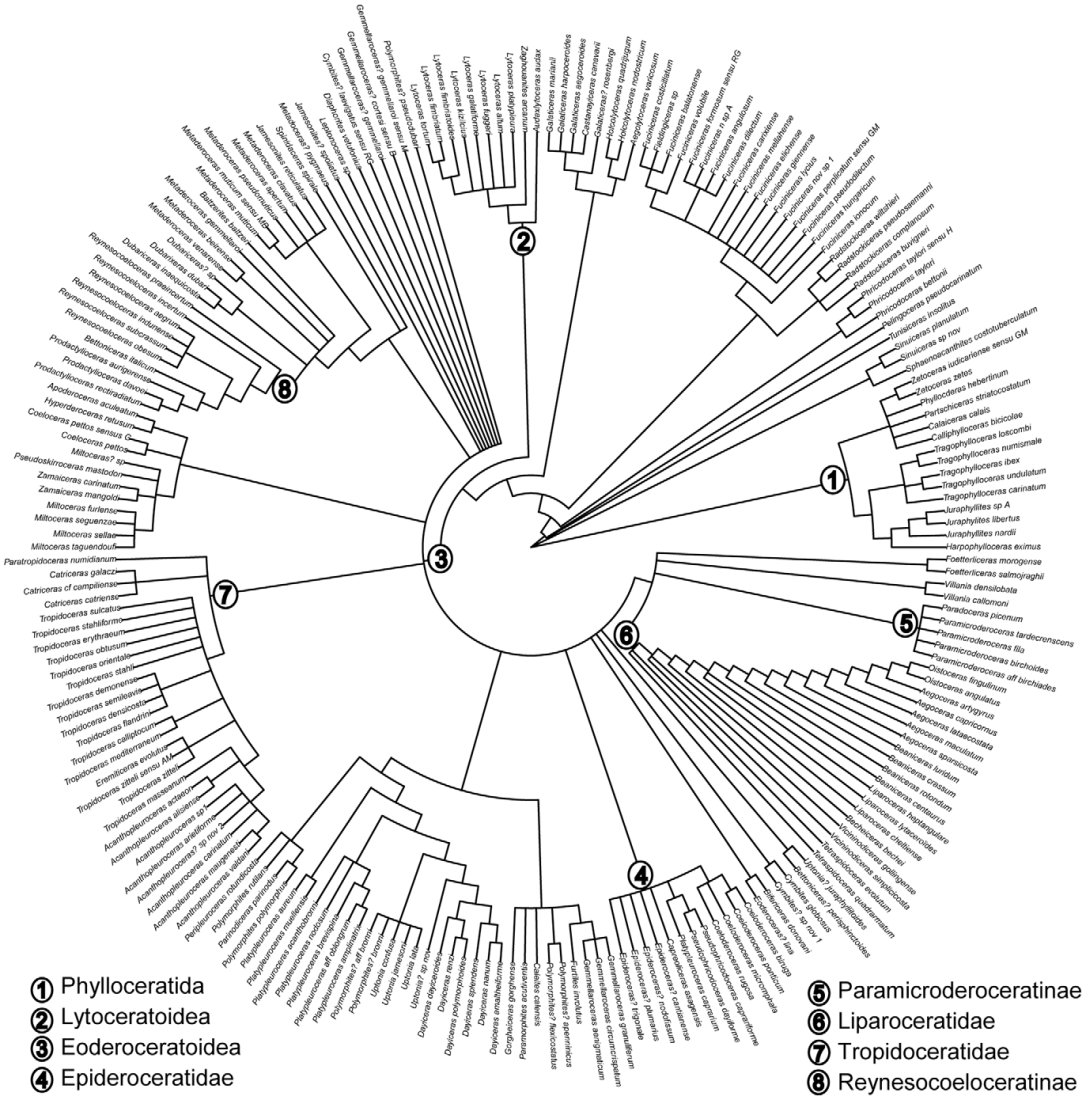
Species-level phylogenetic hypothesis for early Pliensbachian ammonites. Nodes 1 to 8 refer to the clades mentioned in the text.

Out of the 495 nominal species recorded in the literature, Dommergues et al. [32] presented a thoroughly revised dataset of 214 valid ammonite species in each chronozone and subchronozone of the early Pliensbachian interval (estimated mean duration ∼2.2 Myr and ∼0.7 Myr, respectively). Species were regarded as valid after a careful consideration of intra-specific variability, including some possible cases of sexual dimorphism [32], [34]. This dataset covers the western Tethys and adjacent areas, *i.e*., a surface of about 10^7^ km^2^. Here we further introduce extinction data and a phylogenetic hypothesis (Figure 1). It thus extends and updates Dommergues and Meister's [35] work and it is based on the same methodology. This composite tree is a cladistic formalization of the phylogenetic relationships among all well-established Pliensbachian ammonite clades, together with a thoroughly revised positioning of individual species within them (see also [32], [36]–[41]).

In the absence of branch length information, all species are positioned at the same distance from the root, making our tree ultrametric. Distances were obtained after slicing the phylogeny at a regular nodal depth, with the most closely-related species being at a distance of one unit (see [21], [42]–[44] for similar approaches). This comprehensive phylogenetic framework was then decomposed into as many sub-trees as there are different time intervals (3 chronozones and 10 subchronozones) in order to keep only the species actually present in those time spans (Figure S1). The use of ammonites to define biochronological units is not a confounding factor because less than 10% of the species are involved in the definition of these units in our samples and their removal from the dataset does not affect our results.

### Measuring the phylogenetic distribution of extinctions: a brief review

An ideal method for measuring the phylogenetic distribution of extinctions should concomitantly fulfil the following criteria: (1) to be appropriate for binary traits; (2) to take the whole phylogeny into account at once (and not just a single phylogenetic or taxonomic level) and (3) to be independent from tree size, tree balance, phylogenetic resolution, and character prevalence (*i.e*., the overall percentage of extinction), making the measured strength of the phylogenetic signal comparable across datasets.

Initially designed to evaluate spatial autocorrelation for quantitative variables, the Moran's *I* statistic [45] has later been borrowed to test for phylogenetic autocorrelation (*e.g*. [19], [46]). Although this statistic was not initially proposed for binary variables (*contra* criterion 1), it is regarded as a robust approach for detecting taxonomic patterns of extinction risk [16], [20], [21]. It is broadly insensitive to tree size and tree balance [20], but this statistic does not entirely satisfy criteria (3) because of its sensitivity to the percentage of extinction [20] and possibly to the number of taxa [3]. The Moran's *I* statistic can be calculated as follows:



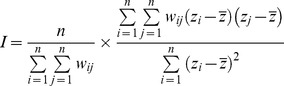



with *n* the total number of taxa, *z* the value of the variable (here extinction or survival), and *w_ij_* the taxonomic or phylogenetic proximity between two taxa (*w_ij_*≥0 and *w_ij_* = *w_ji_*).

Moran's *I* is usually displayed as a taxonomic correlogram in which autocorrelation values are plotted against successive taxonomic or phylogenetic levels [16], [19]–[21], [47]. In this case, *w_ij_* is binary: it takes a value of 1 if taxa *i* and *j* belong to the same level, and equals 0 otherwise. The shape of correlograms provides information on the evolutionary rate of the trait (or combination of traits) under study. A constant rate across the phylogeny produces a correlogram exponentially decaying to 0 toward the basal-most phylogenetic levels and this can be regarded as an evolutionary null model [20], [47]. Departures from this model can thus inform on the phylogenetic conservatism and depth of the signal.

Another, complementary option is to employ the generalized version of Moran's *I* (*sensu* [48], [49]). In this version, *w_ij_* is based on the whole topology of the tree and it actually measures the phylogenetic proximity between taxa *i* and *j*. Its advantage is to provide a single value of phylogenetic autocorrelation for an entire tree (thus satisfying criterion (2)). Following Pavoine et al. [49], we computed *w_ij_* (referred to as *a_ij_* in Pavoine et al. [49]) as the inverse of the product of the number of branches descending from each node in the path connecting *i* and *j*. This approach is analytically and conceptually adequate for our dataset because it enhances the power of Moran's test and is applicable to not fully-resolved trees.

It must be noted that in both versions of Moran's *I*, the calculated values are identical when either extinctions or survivals are investigated. This arises from the mathematical properties of this index and not necessarily from a phylogenetically symmetrical pattern of extinctions and survivals (see Figure S2 for a commented example). In turn, Moran's *I* actually measures an overall phylogenetic signal rather than the phylogenetic clustering of extinctions *per say*.

**Figure 2:**
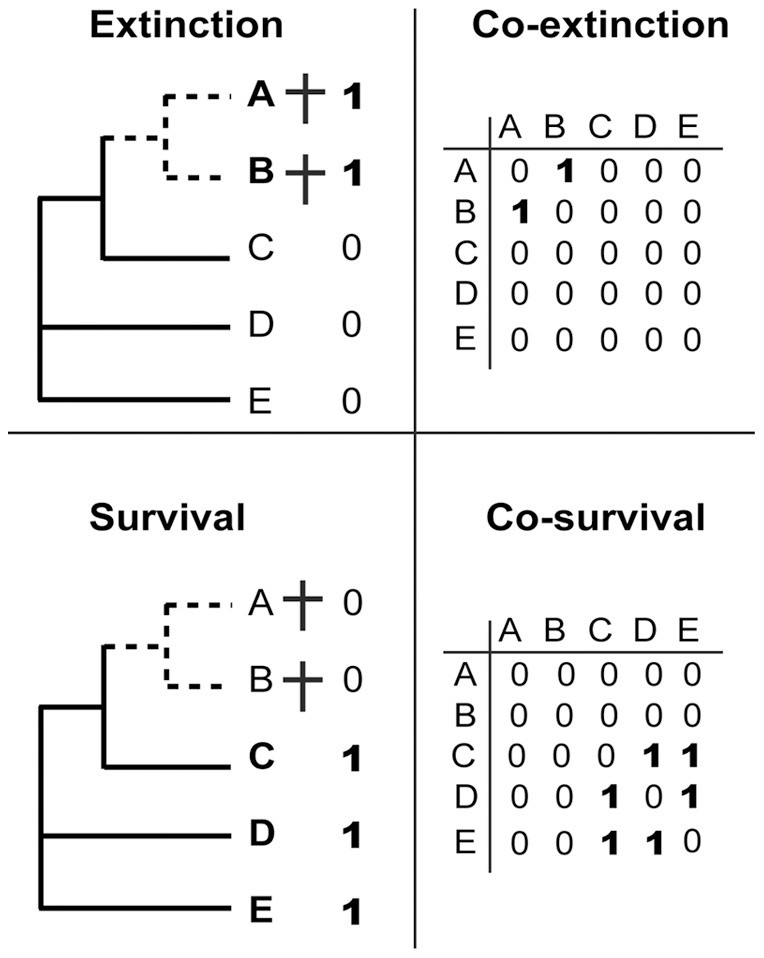
Co-extinction and co-survival matrices for a same theoretical phylogeny of five species (A–E) among which species A and B become extinct (daggers). The two matrices differ in structure when either extinctions or survivals are coded by “1”.

Using fossil data, Roy et al. [3] used the Pearson's product-moment correlation coefficient *r* to investigate the phylogenetic pattern of extinctions among bivalves for a single taxonomic level (genera within families). In order to investigate the phylogenetic signal through all taxonomic or phylogenetic levels, we propose here to use this method with correlograms in a manner similar to Moran's *I*. These two indices are related because they correspond to a covariance/variance ratio that always (Pearson's *r*) or usually (Moran's *I*) takes values in the interval [−1, +1] (*e.g*. [50], [51]). However, Pearson's *r* is not an autocorrelation index *per say*. Here it measures the correlation between two similarity matrices, one with taxonomic information (taxa in the same clade or not) and the other with co-extinction status (taxa co-extinct or not) [3]. This approach thus tests the correlation between two binary variables. In that particular case, Pearson's *r* corresponds to the Phi coefficient or Pearson's Phi (noted *ϕ* or *r*_Φ_) that is specifically designed for two binary variables, and is also equal to the Spearman rank-order correlation coefficient r_s_ even when the latter is corrected for ties (*e.g.* [50], [52]). Hereafter we shall refer to the Pearson's *ϕ* coefficient to emphasize the binary state of the variables. This method therefore satisfies criterion (1) but it does not take the whole phylogeny into account (*contra* criteria (2)). Its major advantage is that it distinguishes extinction and survival patterns, notably because the co-extinction matrix differs in structure from its survival counterpart (Figure 2).

More recently, Fritz and Purvis [23] derived an index, *D*, for measuring the phylogenetic signal of binary traits that fulfills nearly all the above-mentioned criteria. Their proposal builds on previous studies that used the sum of sister-clade differences for assessing phylogenetic patterns of extinction risk [10], [22]. Indeed, the *D* statistic scales the sum of sister-clade differences (Σ*d*_obs_) with those expected under a random (Σ*d*_r_) model and a Brownian (Σ*d*_b_) evolutionary model. These models are respectively generated after 1000 permutations of extinctions and 1000 evolutionary simulations under a Brownian motion (see [23] for details):



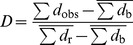

[23]

**Figure 3:**
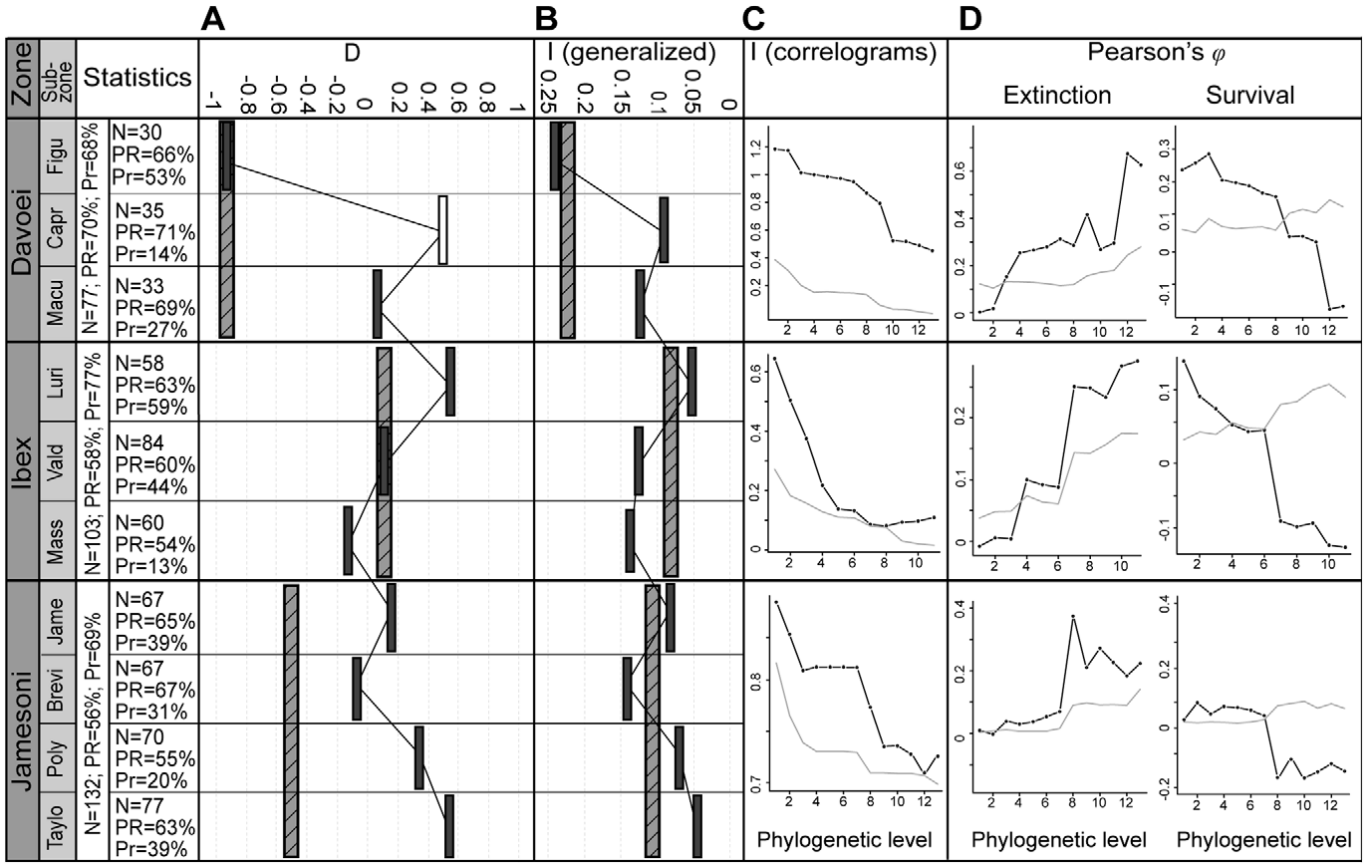
Phylogenetic distribution of ammonite extinctions and survivals. The first column provides the associated statistics for individual time bin (N: species richness, PR: phylogenetic resolution, Pr: prevalence of extinction). (A) and (B) show respectively Fritz and Purvis *D* and the generalized Moran's *I* for all chronozones and sub-chronozones of the early Pliensbachian. Vertical hatched bars and grey bars represent significantly non-random values at the chronozone and subchronozone level, respectively. The white bar corresponds to a *D* value that is not significant. Moran's *I* and Pearson's *φ* correlograms for chronozones are given in (C) and (D), respectively (black line). The thin grey line represents the upper 95% limit of the null model (phylogenetically random extinctions). The deeper the phylogenetic levels, the higher their values on the x-axis. Abbreviations of subchronozones: Taylo: Taylori, Poly: Polymorphus, Brevi: Brevispina, Jame: Jamesoni, Mass: Masseanum, Vald: Valdani, Luri: Luridum, Macu: Maculatum, Capr: Capricornus, Figu: Figulinum.

A phylogenetically random binary trait yields a *D* value of 1, a trait with a Brownian (clumped) phylogenetic pattern has a *D* equal to 0, whereas values below 0 correspond to extremely clumped patterns. Note that by construction, *D* integrates information of both extinctions and survivals. This statistic is particularly suited for investigating phylogenetic patterns in successive time intervals because its scaling permits a direct comparison of the strength of the signal among datasets, regardless of trait prevalence, tree size and tree shape [23]. Nonetheless, Fritz and Purvis [23] mentioned that *D* may have practical limitations for small trees (<25 tips), for trees combining a relatively small size (<50 tips) and extreme levels of trait prevalence, and for trees with a poor resolution (<70%, as measured as the ratio of the number of nodes in the observed tree to the number in a completely resolved tree). In such cases, variation in *D* estimates is high and its statistical power reduced. Our dataset is mostly unaffected by these problems (Figure 3), but the rather low phylogenetic resolution (from 54% to 71%) suggests that significant *D* values will have to be interpreted cautiously.

In our study, statistical significance was tested against null models obtained after 1000 (Moran's *I*) or 5000 (Pearson's *ϕ*) permutations of extinct species (see also [3], [20]). In other words, we randomly permuted the trait (extinction and survival) at the tips of the original phylogenetic topology. This simulates the null hypothesis in which extinctions are randomly distributed in the phylogeny (i.e. there is no phylogenetic autocorrelation in the data), and we used one-tailed tests with *α* sets at 5%. The *D* statistic was further tested against a Brownian distribution [23]. All indices and tests were computed in the statistical computing language R [53]. We generated Moran's *I* correlograms and null models with our own R program. Phylogenetic proximity for the generalized Moran's *I* was computed with the *Maymat* R function provided by Pavoine et al. [49]. The *D* statistic was computed with Fritz and Purvis' function phylo.d [23], which is part of the CAIC package [54]. We used the APE package [55] to read phylogeny data and to calculate phylogenetic distances.

We applied all these methods to early Pliensbachian ammonites at the chronozone and subchronozone temporal scales.

## Results

Values of *D* and their associated probabilities show that the overall phylogenetic pattern is significantly non-random (*p_rand_* ≤0.003) in the three chronozones of the early Pliensbachian (Figure 3A, hatched bars). The *D* statistic further indicates that this pattern cannot be distinguished from a Brownian model (Jamesoni *p_brown_* = 0.57; Ibex *p_brown_* = 0.39; Davoei *p_brown_* = 0.99). It also reveals that the phylogenetic clustering was particularly high in the Davoei chronozone (*D* = −0.85) and much lower in the Ibex chronozone (*D* = 0.14). Note that the generalized Moran's *I* yields the same pattern (Figure 3B, hatched bars). Moran's *I* correlograms are consistent with these results and further show that the phylogenetic pattern is significant at all phylogenetic levels (Figure 3C). Moreover, Moran's *I* correlograms differ among the three chronozones. The correlogram for Ibex shows a concave decay, whereas Jamesoni and Davoei correlograms indicate a marked increase in clustering for medium phylogenetic levels (from level 4 to level 9, corresponding broadly to families). This hump-shaped pattern results from the significant clustering of both extinctions and survivals that superpose themselves at these phylogenetic levels. Indeed, Pearson's *ϕ* correlograms show that the patterns of extinction and survival are neither identical nor symmetrical. Extinctions are significantly clustered at nearly all phylogenetic levels, except near the tips of the tree where only survivals are significantly clumped (Figure 3D).

The phylogenetically non-random pattern is also pervasive at the temporal resolution of the subchronozone. This major result remains unchanged when *α* is set at 1% or when the conservative Bonferroni correction is applied to account for multiple comparisons. Interestingly, although *D* and the generalized Moran's *I* are mathematically different, they vary similarly through time (Figure 3A and 3B). These variations are consistent with the patterns obtained with correlograms (Figure S3). Note that *D* and *I* values are not correlated with extinction intensity (Spearman rank-order correlation coefficient *r_s_* = −0.177, *p* = 0.624 for *I*, *r_s_* = 0.170, *p* = 0.638 for *D*), as expected from both methodological and empirical investigations ([3], [23], and unpublished simulations by C.H.). The only *D* value that does not significantly differ from random is found for the Capricornus subchronozone (*p_rand_* = 0.105). The combination of a small tree size (35 species) and a low percentage of extinction (14%) in that time interval might be responsible for the lack of significance. More generally, the characterization of *D* can be ambiguous in some instances because of its dual comparison with random and Brownian distributions. This is the case for the Taylori and Luridum subchronozones, for which *D* is equal to 0.57 and it differs significantly from both distributions (*p_rand_* = 0.032, *p_brown_* = 0.008 for Taylori; *p_rand_* = 0.014, *p_brown_* = 0.030 for Luridum), whereas it cannot be distinguished from those two distributions in the Capricornus subchronozone (*p_rand_* = 0.105, *p_brown_* = 0.286). This ambiguity for *D* values close to 0.5 arises because the Brownian and random distributions, respectively centered on 0 and 1, either overlap or not. Note that these three particular cases correspond to the highest *D* values, that is, the weakest phylogenetic strength of the signal in our dataset. Figure 3A further indicates that *D* values obtained for the Jamesoni and Davoei chronozones do not reflect those of their respective subchronozones. In turn, it suggests that the strength of the phylogenetic signal cannot be inferred from one temporal scale to another.

## Discussion

Our results show that the fate of early Pliensbachian ammonites was phylogenetically patterned. Pearson's *ϕ* further reveals that this signal is mainly due to significantly clumped extinctions. Because the character states “extinction” and “survival” are complementary, their respective phylogenetic patterns are related. When extinctions are extremely clumped phylogenetically, so are survivals. In such a case the statistical pattern is straightforward and is significant for both evolutionary destinies. However, significance might differ between extinction and survival patterns when the clustering is less pronounced. In this frequent situation, we recommend the use of Pearson's *ϕ* correlograms to dissect the overall phylogenetic signal into its extinction and survival components. This complements the use of Moran's *I* and *D* indices that encapsulate both aspects at once and for an entire phylogenetic tree. Moran's *I* correlograms permit to investigate the overall signal at successive phylogenetic levels, whereas Fritz and Purvis' *D* enables to compare the strength of the signal across datasets because it is insensitive to trait prevalence and tree size and shape [23]. This strongly argues for a joint use of these quantitative indices because they highlight different facets of a same phenomenon and they answer different questions.

Pearson's *ϕ* correlograms suggest that the clustering of extinctions was phylogenetically deep for early Pliensbachian ammonites, whereas the opposite situation prevails for survivals. This profound phylogenetic conservatism of extinctions is particularly marked for the Eoderoceratoidea, in contrast with the long-ranging Lytoceratoidea and Phylloceratida. Within the Eoderoceratoidea however, the pervasive phylogenetic clustering is not properly speaking conservative as it affects small clades that took over each other through time (*e.g*., the Epideroceratidae and the clade gathering Paramicroderoceratinae and Liparoceratidae in the Jamesoni chronozone, the Tropidoceratidae in the Ibex chronozone, or the Reynesocoeloceratinae in the Davoei chronozone). Overall, the maintenance of major ammonite lineages results from two alternative strategies. Clades have either a few long-ranging species or many successive short-range species whose evolutionary dynamism compensates for –or is fuelled by– their clustered extinctions. This scale-dependent phylogenetic conservatism is superposed to the maintenance of a significant clustering of extinction for all temporal intervals in the early Pliensbachian. Such a temporal maintenance occurred despite the environmental and biotic changes documented for that time span. This includes a significant warming of seawater temperatures (about 4 °C) during the Davoei chronozone [26], [27], a sudden bloom of ammonite richness in the Valdani subchronozone [32], and the high and low origination rates characterising the Ibex and Davoei chronozones, respectively [32], [33].

Geography may be a major confounding factor when investigating the phylogenetic autocorrelation of extinctions (*e.g.* [2], [9], [56]). However, for our dataset, preliminary investigations on palaeogeographical maps show that closely-related species becoming extinct or surviving were geographically scattered over the studied area. This is corroborated by other observations. For example, the strongest phylogenetic clustering of extinctions occurs in the Davoei chronozone (Figure 3), an interval whose salient distributional feature is the significant reduction in ammonite endemism ([57], and unpublished data).

Our study documents an evolutionarily volatile clade whose extinctions were significantly clumped phylogenetically throughout a 6.6 Myr-long interval. We thus rally to other authors who advocate methods correcting for phylogenetic autocorrelation when exploring biological traits involved in extinction or survival (*e.g.* [5], [8], [12], [23], [58]). The identification of such traits for early Pliensbachian ammonites is difficult, but they are certainly associated to their mode of life in shallow epicontinental seas. Alternatively, long-ranged ammonite species are phylogenetically clustered within Lytoceratoidea and Phylloceratida, and their habitat, in the vicinity of oceanic basins, may have buffered them from environmental variations.

Finally, our work complements previous investigations as it is intermediate between neontological studies on extinction risks (*e.g.* [8], [21], [23]) and large-scale investigations on past extinctions [3]. However, much work remains to be done if we are to understand how the phylogenetic distribution of extinctions responds to environmental crises and to transitions in extinction regimes through time and across clades.

## Supporting Information

Figure S1 **Ammonite phylogenetic trees for the 3 chronozones and 10 subchronozones of the early Pliensbachian.** Species in red are those becoming extinct during the interval.
(PDF)

Figure S2 **Application of Moran**'**s** ***I*** **to extinction and survival patterns.** A: Simple theoretical phylogenetic hypothesis for five species (A–E), among which species A and B become extinct in a same time interval (daggers); B: Vector corresponding to the coding of either extinctions or survivals as used by the Moran's *I.* Note that only this vector differs between extinctions and survivals, the **W** matrix is the same; C: Moran's *I* will take the same value for both survivals and extinctions due to the mathematical properties of this index. Similarly, Moran's *I* could not distinguish this pattern of extinction from one in which species C, D and E would become extinct although the phylogenetic distance between extinct species differ.
(PDF)

Figure S3 **Moran**'**s** ***I*** **and Pearson**'**s** ***ϕ*** **correlograms for the 10 subchronozones of early Pliensbachian.** In each graph the grey line corresponds to the upper 95% limit of the null model.
(PDF)
