# Supplementary material for: Deep-Time Phylogenetic Clustering of Extinctions in an Evolutionarily Dynamic Clade (Early Jurassic Ammonites)
Source: PLoS One. 2012 May 25;7(5):e37977. doi: 10.1371/journal.pone.0037977 (PMC3360673; doi:10.1371/journal.pone.0037977)

Figure S1.

Jamesoni chronozone

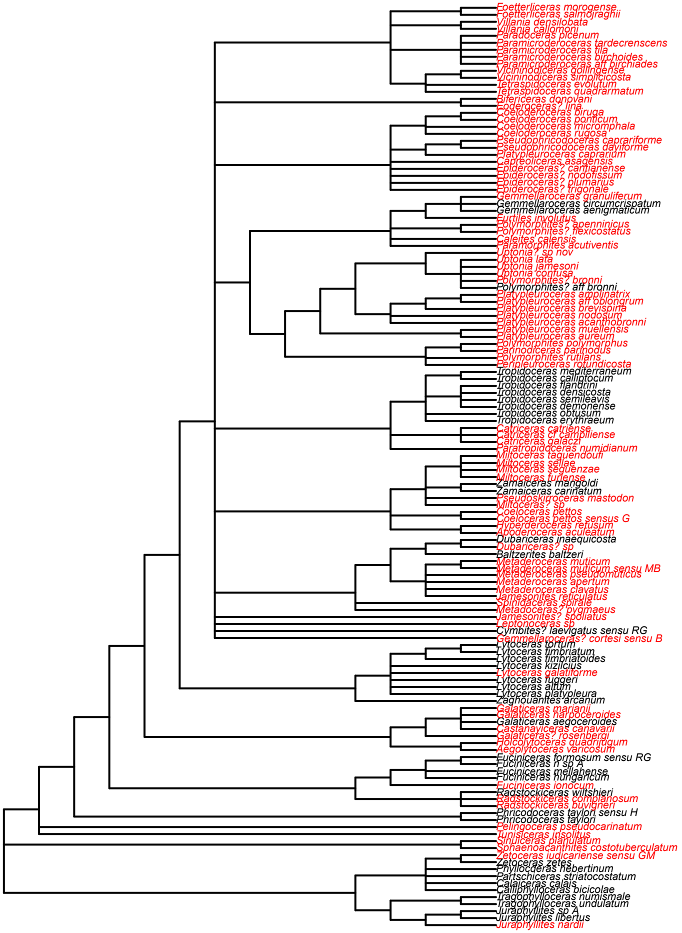

Ibex chronozone

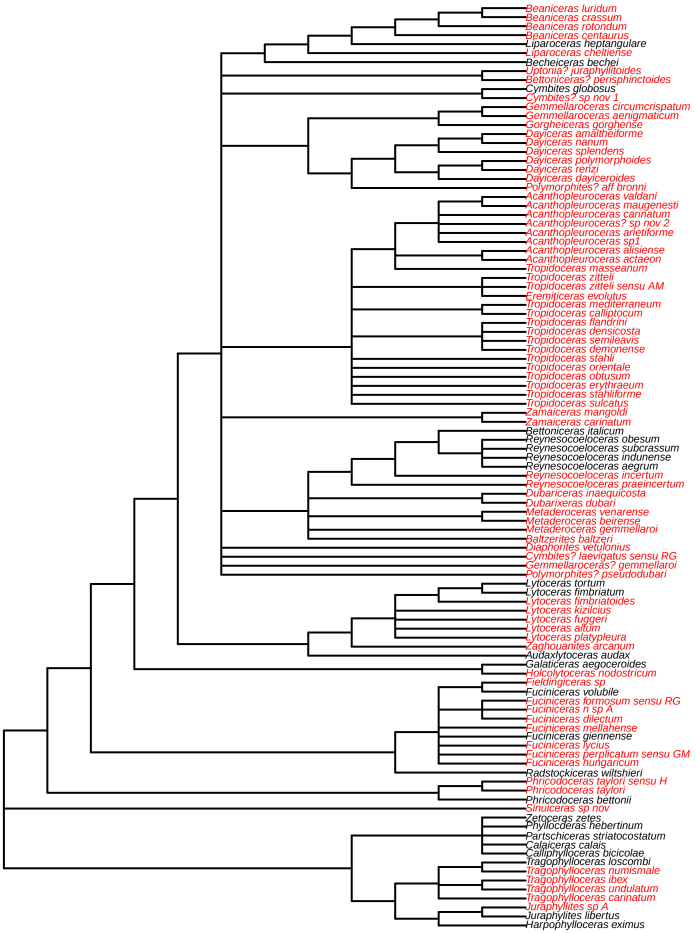

Davoei chronozone

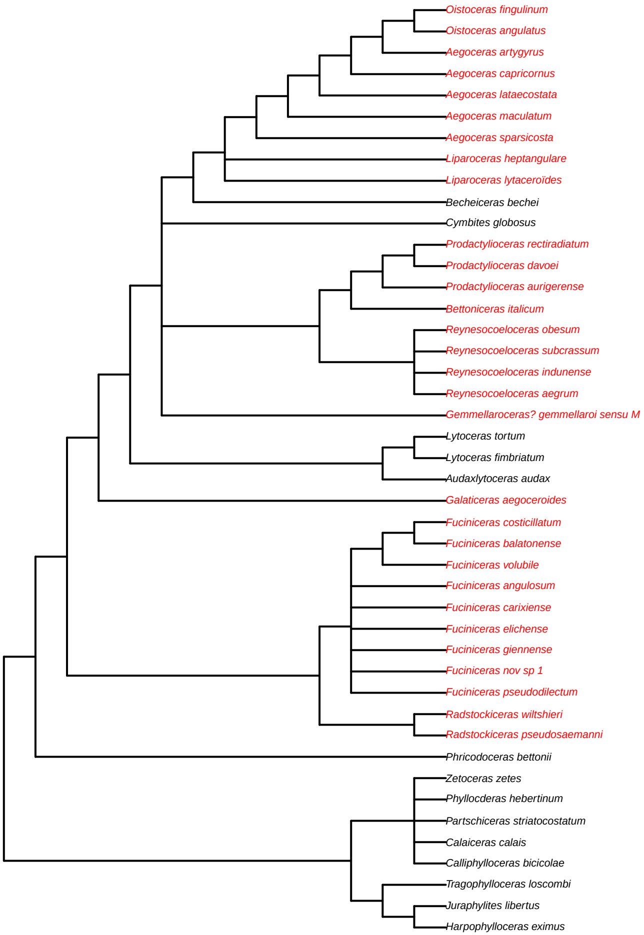

Taylori subchronozone

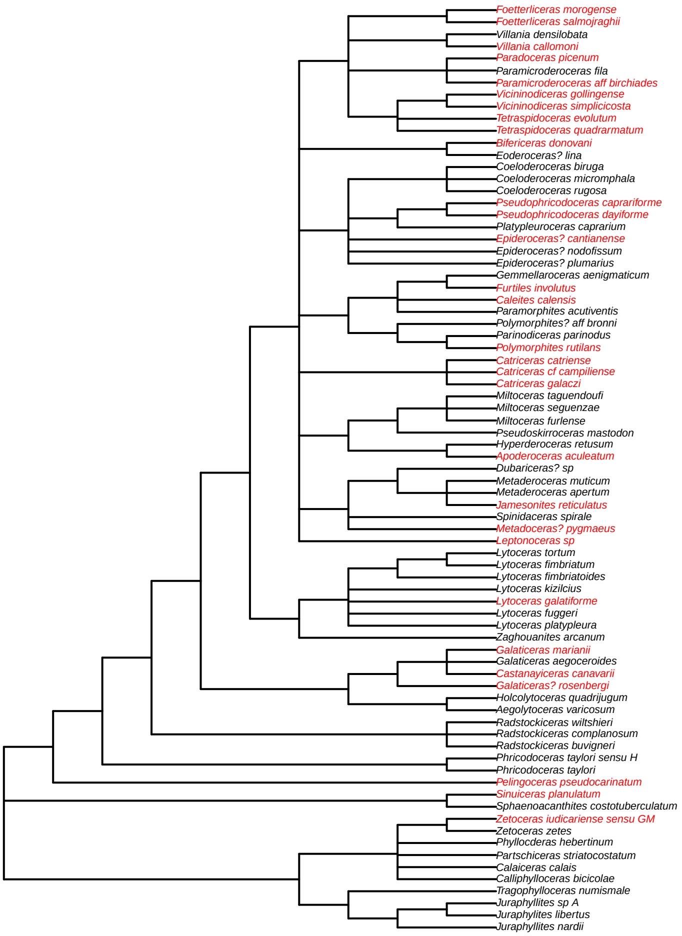

Polymorphus subchronozone

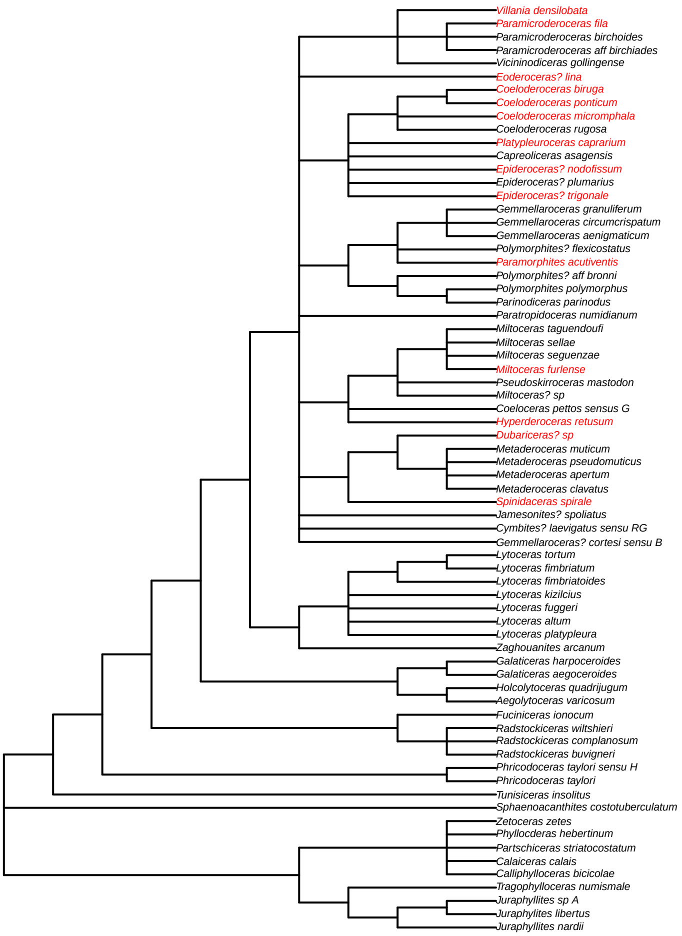

Brevispina subchronozone

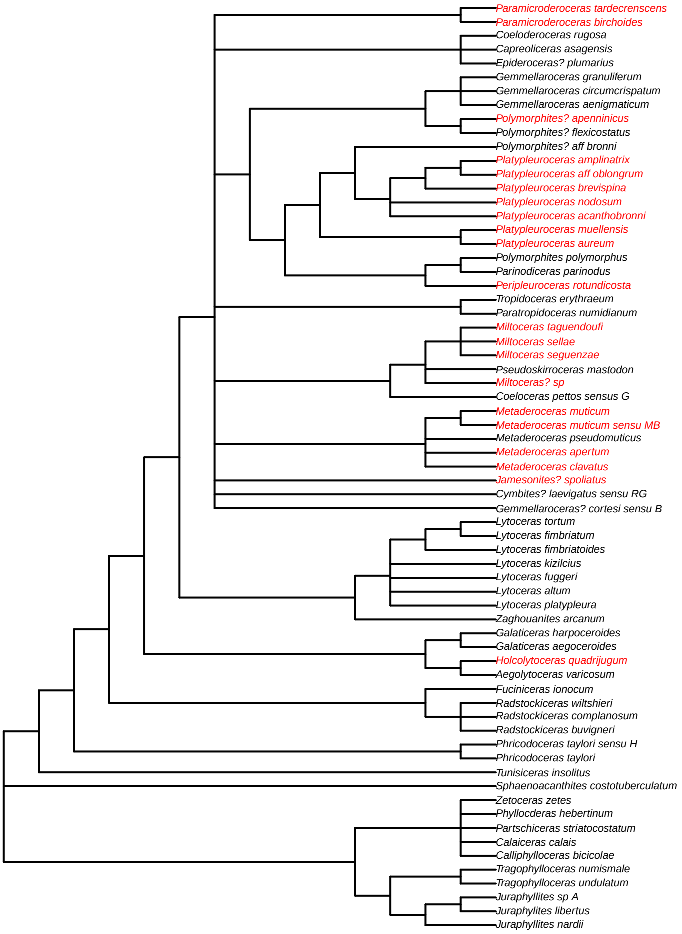

Jamesoni subchronozone

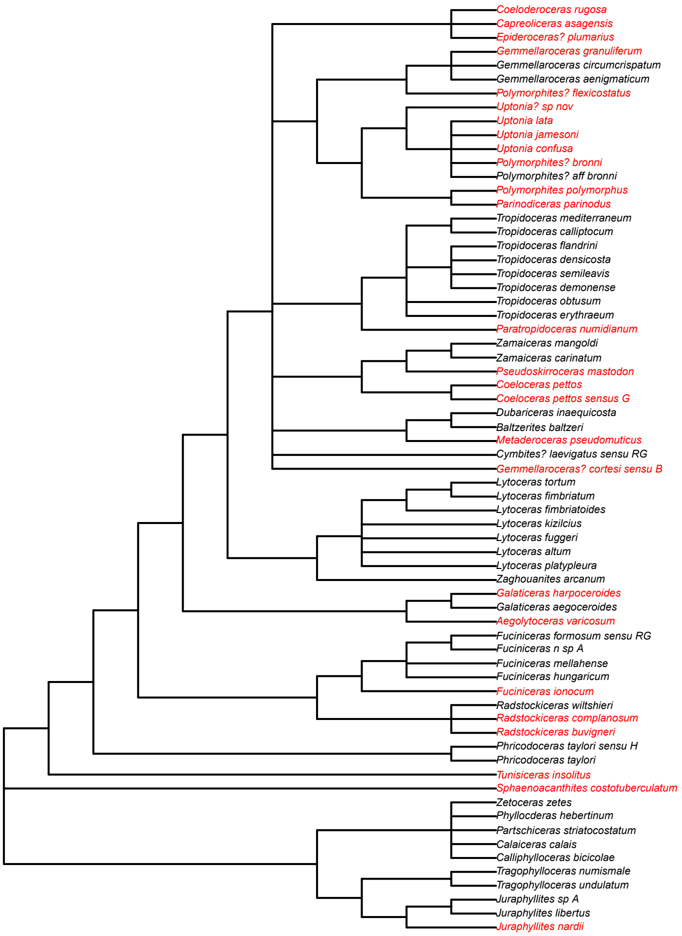

Masseanum subchronozone

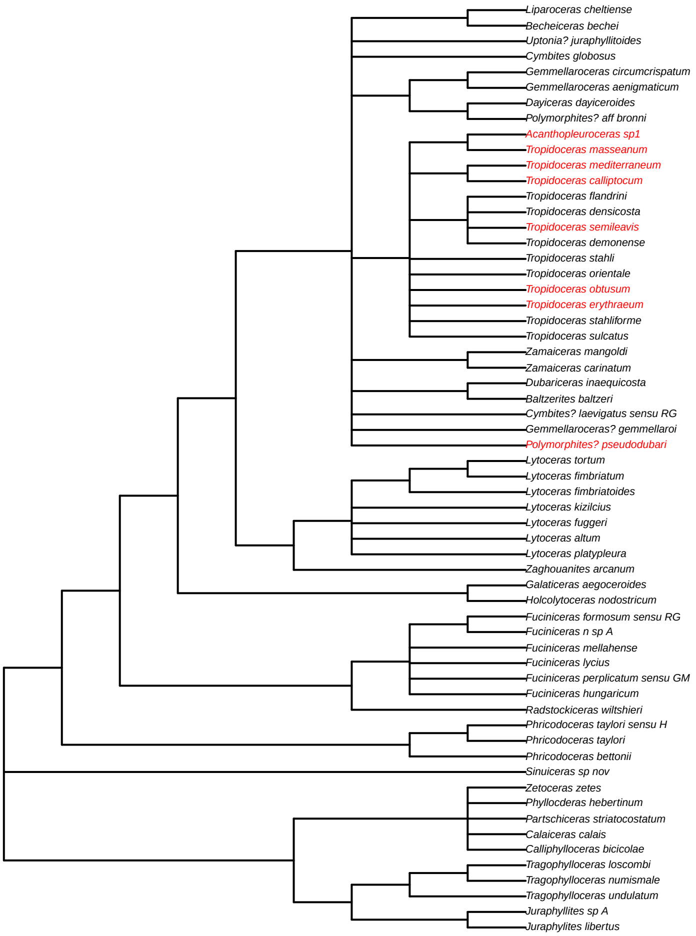

Valdani subchronozone

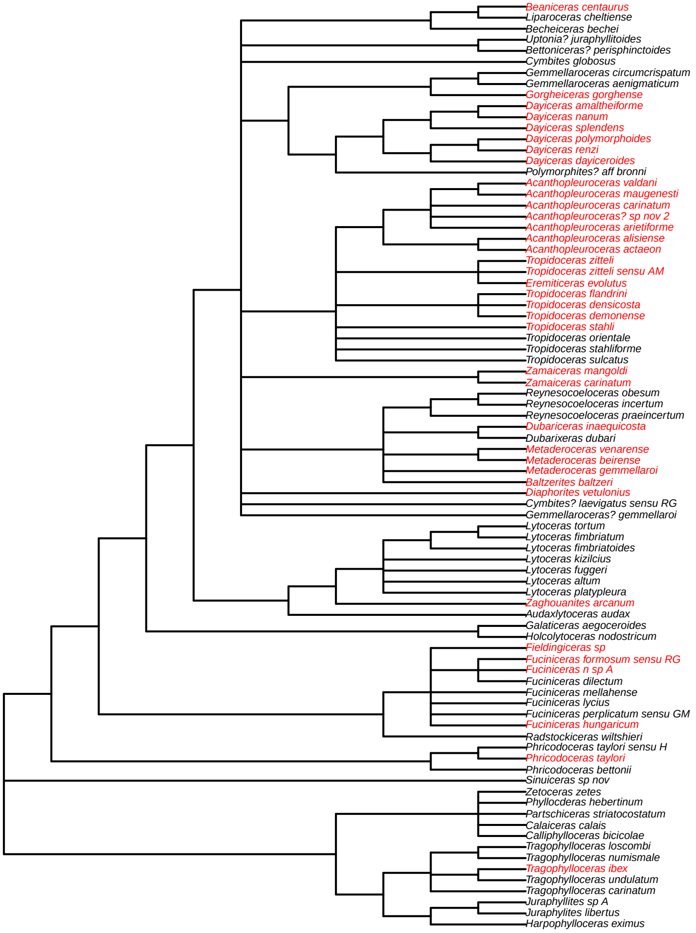

Luridum subchronozone

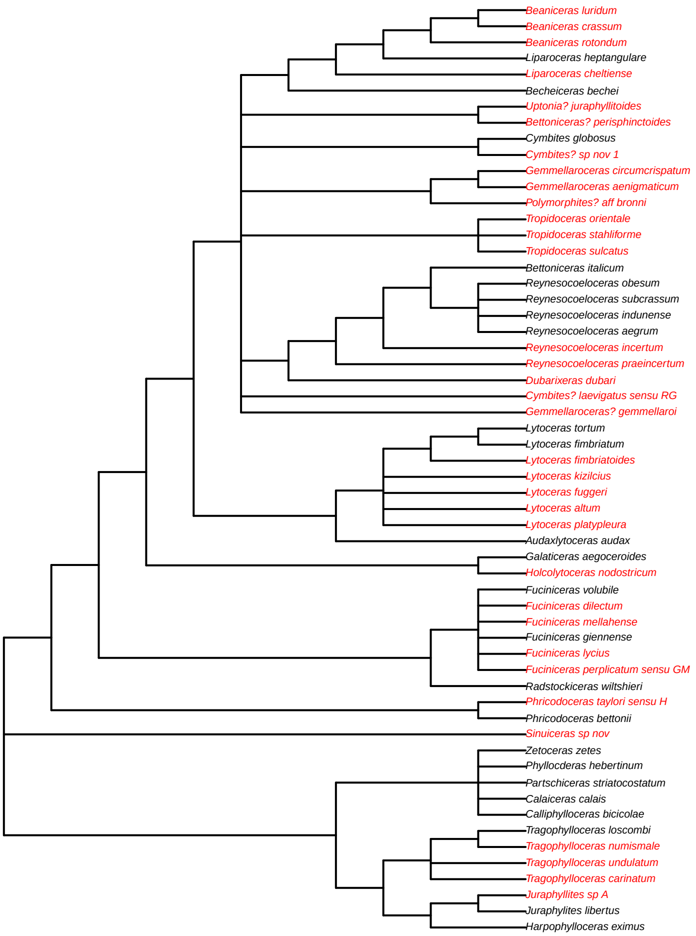

Maculatum subchronozone

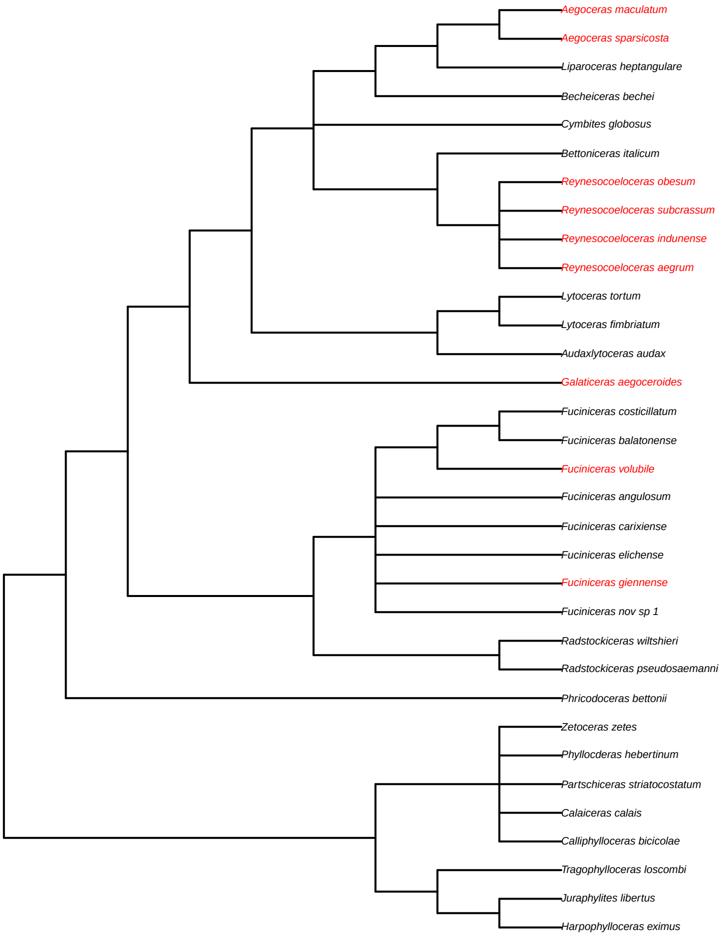

Capricornus subchronozone

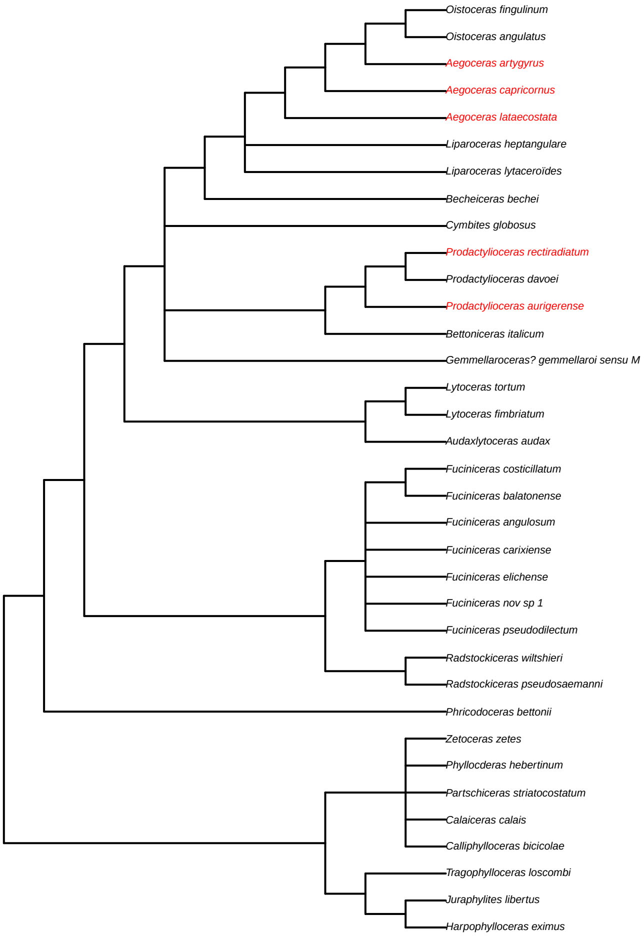

Figulinum subchronozone

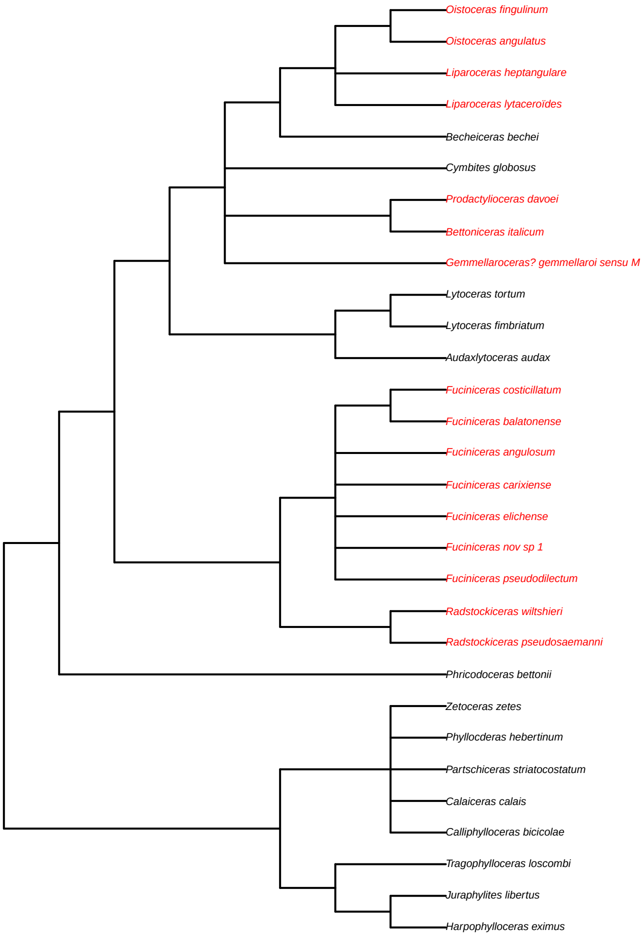

Supplement: Figure S1 — Ammonite phylogenetic trees for the 3 chronozones and 10 subchronozones of the early Pliensbachian. Species in red are those becoming extinct during the interval. (PDF) [file pone.0037977.s001.pdf]
