# Supplementary material for: Deep-Time Phylogenetic Clustering of Extinctions in an Evolutionarily Dynamic Clade (Early Jurassic Ammonites)
Source: PLoS One. 2012 May 25;7(5):e37977. doi: 10.1371/journal.pone.0037977 (PMC3360673; doi:10.1371/journal.pone.0037977)
Supplement: Figure S2 — Application of Moran's I to extinction and survival patterns. A: Simple theoretical phylogenetic hypothesis for five species (A–E), among which species A and B become extinct in a same time interval (daggers); B: Vector corresponding to the coding of either extinctions or survivals as used by the Moran's I. Note that only this vector differs between extinctions and survivals, the W matrix is the same; C: Moran's I will take the same value for both survivals and extinctions due to the mathematical properties of this index. Similarly, Moran's I could not distinguish this pattern of extinction from one in which species C, D and E would become extinct although the phylogenetic distance between extinct species differ. (PDF) [file pone.0037977.s002.pdf]

Figure S2:

A

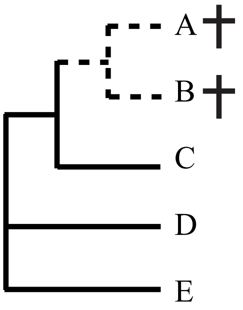

B

Extinction coded by 1	Survival coded by 1
**1**	0
**1**	0
0	**1**
0	**1**
0	**1**

C

$$I = \frac{n}{\sum_{i=1}^{n}\sum_{j=1}^{n} w_{ij}} * \frac{\sum_{i=1}^{n}\sum_{j=1}^{n} w_{ij}\left(z_i - \bar{z}\right)\left(z_j - \bar{z}\right)}{\sum_{i=1}^{n}\left(z_i - \bar{z}\right)^2}$$

Moran's I for extinctions

$$I_{ext} = \frac{n}{\sum_{i=1}^{n}\sum_{j=1}^{n} w_{ij}} \times \frac{w_{ij}\left[\left(1-\frac{2}{5}\right)\left(1-\frac{2}{5}\right)\right] + w_{ij}\left[\left(1-\frac{2}{5}\right)\left(0-\frac{2}{5}\right)\right]\times 6 + w_{ij}\left[\left(0-\frac{2}{5}\right)\left(0-\frac{2}{5}\right)\right]\times 3}{\left[\left(1-\frac{2}{5}\right)+\left(1-\frac{2}{5}\right)+\left(0-\frac{2}{5}\right)+\left(0-\frac{2}{5}\right)+\left(0-\frac{2}{5}\right)\right]^2}$$

$$I_{ext} = \frac{n}{\sum_{i=1}^{n}\sum_{j=1}^{n} w_{ij}} \times w_{ij}\left[\frac{9}{25}\right] + w_{ij}\left[-\left(\frac{6}{25}\right)\right]\times 6 + w_{ij}\left[\left(\frac{4}{25}\right)\right]\times 3$$

$$I_{ext} = \frac{n}{\sum_{i=1}^{n}\sum_{j=1}^{n} w_{ij}} \times w_{ij}\left[\frac{9}{25} - \left(\frac{36}{25}\right) + \left(\frac{12}{25}\right)\right]$$

$$I_{ext} = \frac{n}{\sum_{i=1}^{n}\sum_{j=1}^{n} w_{ij}} \times w_{ij}\left[-\frac{3}{5}\right]$$

Moran's I for survivals

$$I_{sur} = \frac{n}{\sum_{i=1}^{n}\sum_{j=1}^{n} w_{ij}} \times \frac{w_{ij}\left[\left(0-\frac{3}{5}\right)\left(0-\frac{3}{5}\right)\right] + w_{ij}\left[\left(0-\frac{3}{5}\right)\left(1-\frac{3}{5}\right)\right]\times 6 + w_{ij}\left[\left(1-\frac{3}{5}\right)\left(1-\frac{3}{5}\right)\right]\times 3}{\left[\left(0-\frac{3}{5}\right)+\left(0-\frac{3}{5}\right)+\left(1-\frac{3}{5}\right)+\left(1-\frac{3}{5}\right)+\left(1-\frac{3}{5}\right)\right]^2}$$

$$I_{sur} = \frac{n}{\sum_{i=1}^{n}\sum_{j=1}^{n} w_{ij}} \times w_{ij}\left[\frac{9}{25}\right] + w_{ij}\left[-\left(\frac{6}{25}\right)\right]\times 6 + w_{ij}\left[\left(\frac{4}{25}\right)\right]\times 3$$

$$I_{sur} = \frac{n}{\sum_{i=1}^{n}\sum_{j=1}^{n} w_{ij}} \times w_{ij}\left[\frac{9}{25} - \left(\frac{36}{25}\right) + \left(\frac{12}{25}\right)\right]$$

$$I_{sur} = \frac{n}{\sum_{i=1}^{n}\sum_{j=1}^{n} w_{ij}} \times w_{ij}\left[-\frac{3}{5}\right]$$
