# Supplementary figures and images for: Deep-Time Phylogenetic Clustering of Extinctions in an Evolutionarily Dynamic Clade (Early Jurassic Ammonites)
Source: PLoS One. 2012 May 25;7(5):e37977. doi: 10.1371/journal.pone.0037977 (PMC3360673; doi:10.1371/journal.pone.0037977)

Figure S3 :

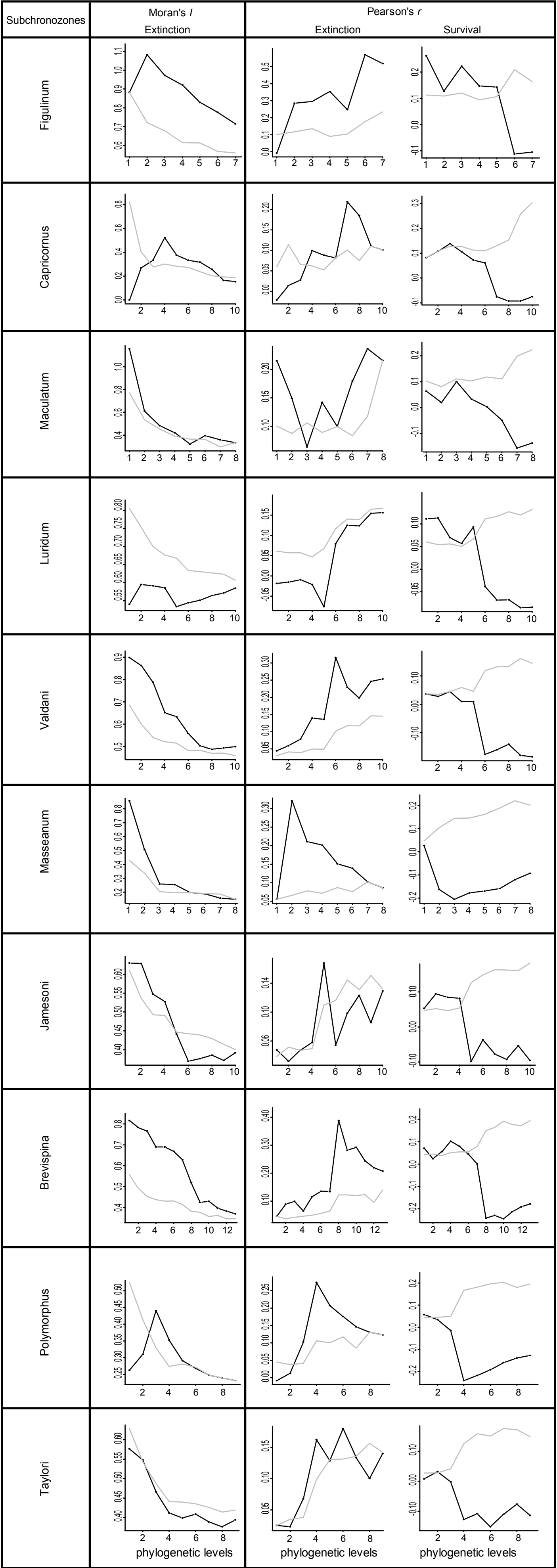

Supplement: Figure S3 — Moran's I and Pearson's ϕ correlograms for the 10 subchronozones of early Pliensbachian. In each graph the grey line corresponds to the upper 95% limit of the null model. (PDF) [file pone.0037977.s003.pdf]
